# Vitamin B6 Neonatal Toxicity

**DOI:** 10.1155/2022/3171351

**Published:** 2022-12-06

**Authors:** Andrea Guala, Giulia Folgori, Micaela Silvestri, Michelangelo Barbaglia, Cesare Danesino

**Affiliations:** ^1^SOC Pediatrics, Castelli Hospital, Verbania, Italy; ^2^Post-graduate School in Pediatrics, University “Piemonte Orientale”, Novara, Italy; ^3^Department of Molecular Medicine, University of Pavia, Pavia, Italy

## Abstract

Vitamin B6 is a micronutrient required by the body. It acts as a coenzyme in biochemical reactions. Vitamin B6 toxicity is not caused by the intake of food-based sources. The few reported cases of vitamin B6 toxicity are always caused by overdosing of nutritional supplements. Chronic toxicity typically occurs with peripheral neuropathy such as paraesthesia, ataxia, and imbalance, paradoxically mimicking vitamin B6 deficiency. However, the prognosis is favorable, and symptoms usually show improvement once excessive vitamin B6 levels return to the physiological range. We report a newborn presenting with diffuse tremor at birth, interpreted as secondary to the mother's intake of high doses of a supplement containing vitamin B6 during pregnancy and breastfeeding. As expected, the newborn's serum levels of vitamin B6 were high. The tremors disappeared when the maternal supplement was stopped.

## 1. Introduction

**Figure 1:**
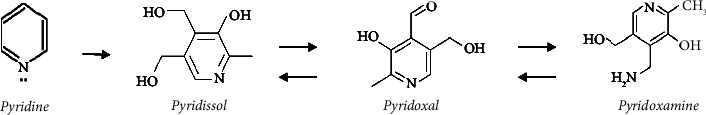
Chemical structure and derivation of the molecules with vitamin B6 action [20].

Vitamin B6 is an essential water-soluble micronutrient, and its requirement in human adults ranges from 0.4–1.3 mg/day [1] to 1.5–1.8 mg/day [2]. Vitamin B6 refers to multiple chemically similar compounds (pyridoxine, pyridoxal, and pyridoxamine), of which pyridoxine is the most common (Figure 1). Cells use pyridoxal phosphate (PLP), the coenzymatic form of vitamin B6, in many different enzymatic reactions such as neurotransmitter production, amino acids, glucose and lipids metabolism, hemoglobin synthesis and function, and gene expression. Vitamin B6 is widely present in foods (meat, fish, poultry, cereals, vegetables, and fruit), and intake of food-based sources of pyridoxine will not usually cause toxicity. The only reported cases of vitamin B6 toxicity are from chronic supratherapeutic dosing of supplements or excessive iatrogenic assumption [3]. Toxicity typically occurs with peripheral neuropathy like paraesthesia, ataxia, and imbalance, paradoxically mimicking a vitamin B6 deficiency. This sensory neuropathy usually develops at doses of pyridoxine above 1000 mg per day in adults, although it has been reported at doses of less than 500 mg per day in patients taking supplements for several months. None of the studies had sensory nerve damage at daily intakes below 200 mg of pyridoxine per day [4, 5]. The prognosis is usually favorable with symptoms decreasing or resolving when supratherapeutic intake of pyridoxine has been discovered and stopped [6]. We report the case of a newborn with spontaneous tremors whose pregnant and breastfeeding mother had been taking high doses of a dietary supplement containing vitamin B6. The tremors disappeared when the supplement was stopped.

## 2. Case Report

The patient was born after eutocic delivery at 38 weeks of gestation. Birth weight and length were 3150 g and 49 cm, with a head circumference of 33.5 cm. Apgar scores at 1–5 minutes were 9–10. Breastfeeding began approximately 30 min after birth with no formula supplementation. The pregnancy was uneventful, and maternal hypothyroidism was controlled by replacement therapy with levothyroxine sodium, which was the only drug assumed to be under medical control. The mother reported smoking two cigarettes/day during pregnancy but denied alcohol consumption. A few hours after birth, both spontaneous and stimulated tremors involving the four limbs and the chin were noted, associated with skin redness. Tremors were also present while asleep. Clinical examination demonstrated normal vital signs and stable cardiorespiratory parameters. A blood sample revealed borderline hypoglycemia with a blood glucose concentration of 55 mg/dL (reference values 55 mg/dl-65 mg/dl). Infant formula was initiated as a supplement to breast milk to restore optimal glucose concentration.

Normalization of glycemic values was quickly obtained, but tremors persisted, prompting a more extensive evaluation of the patient with capillary hemogasanalysis, blood electrolytes, and vitamins. All results were within normal limits, except for vitamin B6 (102.7 microg/L n.v. 8.7–27.2 microg/L) and vitamin B12 (772 pg/mL n.v. 191–663 pg/mL). The presence of several drugs, such as cocaine, was also excluded.

Consultation with a childhood neuropsychiatrist excluded jitteriness [7]. Brain ultrasound and electrocardiography (ECG) did not disclose any abnormalities.

The presence of levels of vitamin B6 much higher than normal reference values was an indication to test the parents, and a clear increase in the pyridoxine values in the mother (52.1 microg/ml, n.v. 8.7–27.2) was found, while the father showed normal values.

A maternal dietary assessment disclosed a history of high-dose multivitamin supplements (2 tablets/day) available over-the-counter throughout pregnancy and into lactation (Table 1). The intake of vitamin supplements was immediately stopped, and, in the following weeks, tremors decreased together with the reduction of vitamin B6 levels both in the maternal and in the young patient serums (Table 2).

At two months of age, control electroencephalography (EEG) showed rare biphasic sharp wave anomalies in the left central parieto-temporal center, which were interpreted as a manifestation of neurovegetative hyperexcitability. At the same time, a childhood neuropsychiatric consultation described mild autonomic instability in an infant with age-appropriate neuromotor development.

Control EEG and ECG at 6 months of age were normal, and all parameters of physical growth were age-appropriate.

## 3. Discussion

In general, vitamin and mineral supplementation are common habits, aiming to prevent cardiovascular and oncologic diseases and, potentially, obtain mental and physical benefits [8–11], but their true efficacy is still not evident or well demonstrated. Similarly, supplementation during pregnancy has been studied in relation to both maternal and fetal wellness [12, 13].

The only clear evidence for vitamin supplementation, based on its efficacy in the prevention of neural tube defects, has been obtained for folic acid in the periconceptional period.

Maternal diet, vitamin, and mineral supplementation in the postpartum maternal diet can modify the nutrient composition of colostrum and breast milk [14, 15].

Vitamin B6, an essential water-soluble vitamin that acts as a coenzyme in many biochemical reactions, a chronic iatrogenic overdose of vitamin B6, can cause neurological toxicity. The mechanism and specific contribution of the various B6 vitamins to neurological toxicity are largely unknown, but it is likely that they are mediated by inhibition of pyridoxal-5-phosphate-dependent enzymes [3].

Pyridoxine, the inactive form of B6, competitively inhibits pyridoxal-5′-phosphate, leading to symptoms of toxicity that mimic those of vitamin B6 deficiency [4]. Clinical history and physical examination are essential to identify patients in whom a potential drug toxicity is present [16], thus directing towards specific blood tests, including vitamin B6. In case of confirmation of vitamin B6 level well above reference values, the intake of drugs or supplements, identified as the cause of neurological symptoms, must be immediately stopped. Consequently, in the following weeks, the toxicity levels of vitamin B6 will progressively decrease with the complete normalization of the clinical picture.

For the patient we report, anamnestic data suggest that the high levels of vitamin B6 in the fetus are more likely due to transfer in the womb. In fact, the mother referred to a prolonged assumption of dietary supplements (as self-medication), continuing after delivery, which stopped only after the identification of an increased level of the nutrient. The transfer of vitamin B6 from mother to fetus during pregnancy has been known for a long time, as has a possible maternal vitamin B6 deficiency related to the active diaplacental transport and the request for vitamin B6 supplementation [17]. Similarly, several authors studied the relation between Vitamin B6 intake in the mother, the levels in breast milk, and the effects on some behavioral abnormalities in children [18, 19].

In our case, vitamin B6 levels, although clearly higher than normal values, were below the levels previously reported as being able to induce clinically evident toxicity [4, 5].

Available evidence and the list of supplements that the mother has assumed suggest that vitamin B6 is reasonably the only nutrient that could be related to the clinical symptoms observed.

Hadtsteinand and Vrolijk [2] hypothesize that PDXK (pyridoxal kinase) inhibition, among others, by vitamin B6 availability results in GABA neurotransmission disruption and could be “*the most plausible mechanism of B6 toxicity*.”

The occurrence of vitamin B6 toxicity because of its supraphysiological supplementation might, of course, be associated with the presence of rare genetic variants, and PDXK might be a candidate gene to be investigated.

Our speculation is that some alleles in genes encoding proteins of vitamin B6 metabolism and/or excretion might promote in some subjects an accumulation of vitamin B6 or a greater cellular sensitivity to its action. This would lead to pyridoxine accumulation in the body, with a consequent toxic effect and the development of neurological manifestations.

The limitations of this report are mainly related to the fact that our data were obtained from a single case.

We believe in the importance of this study, which underlines that a widespread assumption of apparently harmless supplements without medical control may cause relevant clinical problems.

The “take home message” is that (i) it is important to remind doctors and nurses caring for pregnant women to inform their patients about the possible existence of clinical risks even in assuming “over the counter” products; (ii) toxic effects of self-medication may be transferred to the fetus; and (iii) information about possible, even if rare, toxic effects should be always plainly reported in the accompanying leaflet.

## Figures and Tables

**Table 1:** Vitamin content of 2 tablets (^dose taken by the mother of the over-the counter supplement during pregnancy);^*∗*^NRV = Nutritional Reference Values.

Vitamin	Two tablet content	^*∗*^NRV%
Vitamin B6	4 mg	286%
Folic acid	400 mcg	200%
Vitamin B12	6 mcg	240%

**Table 2:** Vitamin B6 serum levels found in patient and mother. ^*∗*^serum normal levels (n.v. 8.7–27.2 micrograms/L).

	October 2020	November 2020	December 2020	April 2021
Newborn, serum B6 micrograms/L	102.7^*∗*^	30.8^*∗*^	59.1^*∗*^	24.6^*∗*^
Mother, serum B6 micrograms/L	400 mcg	52.1	22.1^*∗*^	20.6^*∗*^

## Data Availability

No data are available, in addition to data reported in the text.
